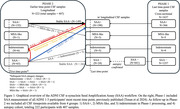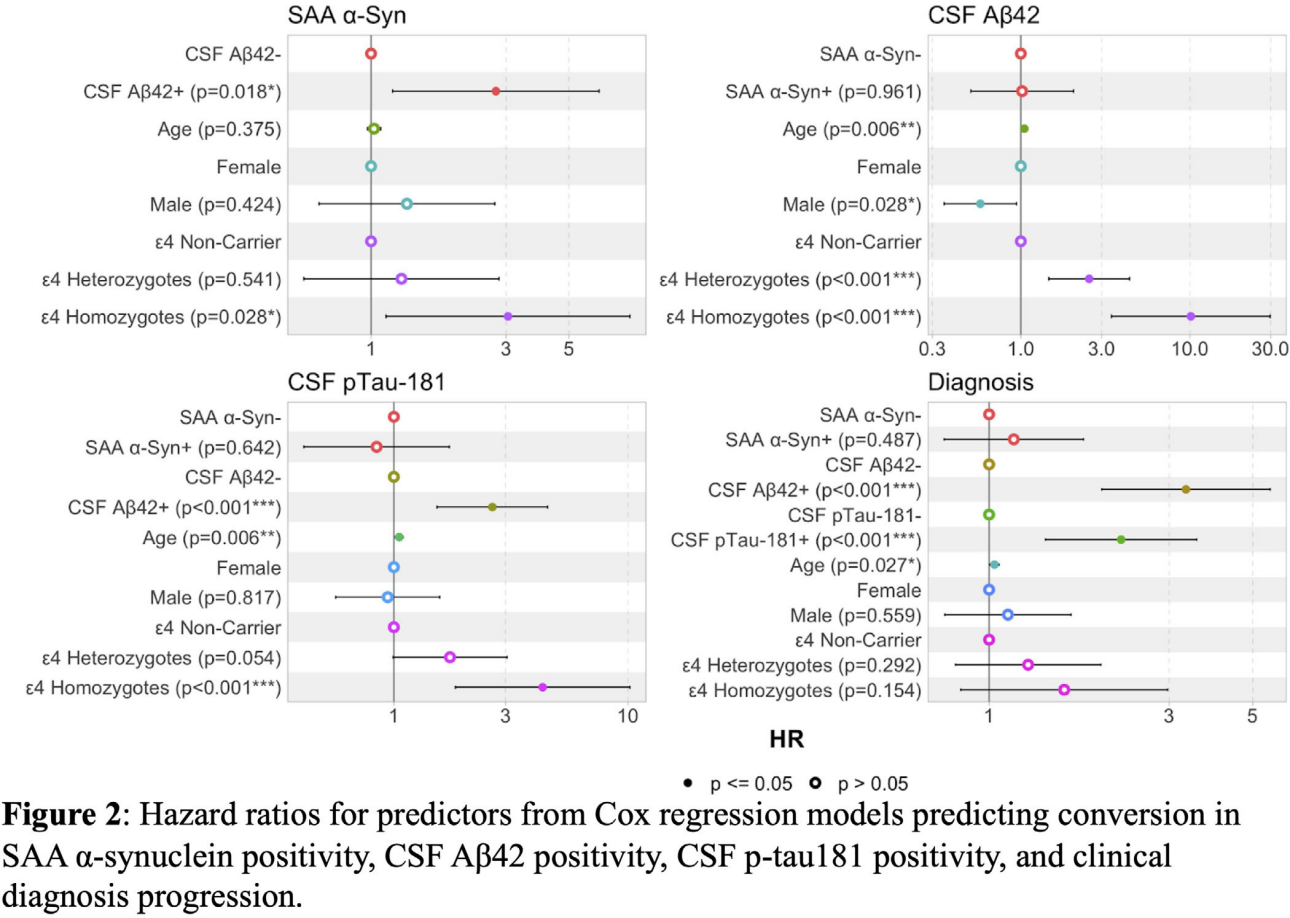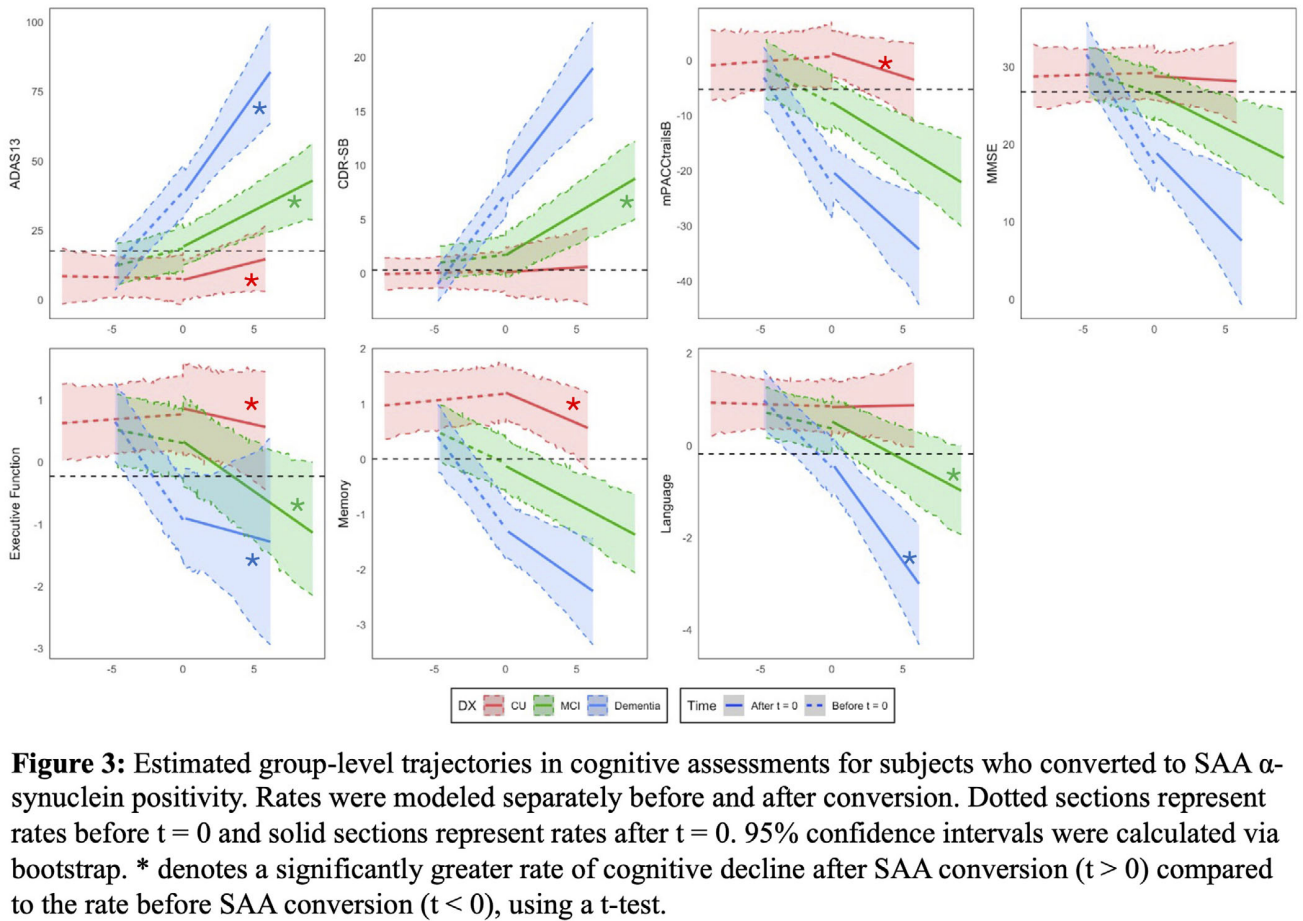# Exploring the Relationship Between Aβ and α‐Synuclein Pathologies: Longitudinal CSF Analysis and Cognitive Decline in the ADNI

**DOI:** 10.1002/alz.095312

**Published:** 2025-01-09

**Authors:** Duygu Tosun, Zachary Hausle, Pamela Zobel‐Thropp, Leslie M. Shaw, Luis Concha, Andrew Singleton, Michael W. Weiner, Cornelis Blauwendraat

**Affiliations:** ^1^ Department of Radiology and Biomedical Imaging, University of California, San Francisco, San Francisco, CA USA; ^2^ Department of Veterans Affairs Medical Center, Northern California Institute for Research and Education (NCIRE), San Francisco, CA USA; ^3^ University of Pennsylvania, Philadelphia, PA USA; ^4^ Amprion, San Diego, CA USA; ^5^ Center for Alzheimer’s and Related Dementias, National Institute on Aging and National Institute of Neurological Disorders and Stroke, National Institutes of Health, Bethesda, MD USA; ^6^ University of California, San Francisco, San Francisco, CA USA; ^7^ National Institute of Neurological Disorders and Stroke, Bethesda, MD USA

## Abstract

**Background:**

AD and Lewy body (LB) disease are characterised by the pathological deposition of Aβ and α‐synuclein, respectively, and represent two of the most common forms of neurodegenerative diseases. Co‐pathologies are commonly identified at autopsy, suggesting crosstalk between Aβ and α‐synuclein during disease progression. CSF α‐synuclein seed amplification assay (SAA) has proven to be a sensitive and specific tool for detecting α‐synuclein seeds as evidence of LB‐pathology. We recently applied SAA cross‐sectionally to the ADNI cohort on the most recent CSF draw (Phase‐1) and looked at LB pathology prevalence (SAA positivity, SAA+) and its association with AD biomarkers and cognition. Here, we expand the ADNI CSF SAA analysis by incorporating longitudinal time‐points (Phase‐2), specifically focusing on SAA+ participants from Phase‐1. We examined the longitudinal dynamics of Aβ, α‐synuclein seeds, and p‐tau181, along with global and domain‐specific cognitive measures in stable SAA+, stable SAA‐, and those who converted to SAA+ from SAA‐.

**Method:**

In longitudinal Phase‐2 study, 407 CSF samples from 222 participants across ADNI‐1‐3 phases were tested with SAA. Cox regression models were performed to assess factors increasing risk for biomarker positivity progression, separately for SAA, CSF Aβ42, and CSF p‐tau181. Additionally, factors contributing to clinical diagnosis progression (CU‐to‐MCI or MCI‐to‐Dementia) were assessed. Changes in cognitive decline rates accompanying SAA conversion were assessed.

**Result:**

Among Phase‐2 participants, 34 individuals progressed from SAA− to SAA+ by their final CSF time‐point (“SAA Converters”; Figure‐1). Conversion to SAA+ was associated with *APOE* ε4 homozygosity, Aβ pathology (Figure‐2), and greater rates of cognitive decline after time of SAA conversion (Figure‐3). SAA status did not impact progression of either Aβ or tau phenotypes, or progression in clinical diagnosis (Figure‐2).

**Conclusion:**

The findings of this longitudinal study demonstrated a significant association between Aβ pathology and onset of α‐synuclein pathology, as measured by SAA. Moreover, presence of SAA‐positivity was linked to accelerated cognitive decline, although greater risk for clinical diagnosis progression was associated with positivity in Aβ42 and p‐tau181 but not SAA‐positivity. These results highlight the interplay between Aβ and α‐synuclein and their impact on disease progression, emphasizing the importance of further investigation into their underlying mechanisms.